# The White City Case

**Published:** 1917-03-10

**Authors:** 


### THE WHITE CITY CASE.

The conclusion of the trial of the men indicted recently at the Central Criminal Court for conspiracy to defeat the provisions of the Military Service Acts suggests several reflections, some pertinent merely to this particular case, others of a more general application. The most obvious criticism of the sentences passed upon the four men who were convicted is that they are extraordinarily lenient, a criticism which was powerfully voiced the next morning in the *Times*, and gains additional point from the Judge's remark that in most countries capital punishment would have been awarded for their offences. It is quite possible that in the case of the two medical men concerned, the Judge was mindful of the fact that their imprisonment will entail also their probable removal from the Medical Register and the certain ruin of their professional careers. This consideration deserves some weight; but does not apply to the two younger defendants, nor does it explain why all four should pass their period of detention in the second division. For the life of us we cannot see why such tenderness should be shown to men who are morally traitors to their country for sordid gain, and have in addition enabled others to be traitors to their country too.

Next, the evidence clearly shows that the War Office, and particularly the authorities of the Army Medical Service, have been much too haphazard in their selection of medical men for such exceedingly important duties as those carried out at the White City by convicts Caley and Dow. Neither of these men appears to have a record which—professionally speaking—is better than third rate; and to put such men, without even the formality of commissioning them (a small, but not a negligible, attempt at a guarantee) to such very responsible work is merely one of those War Office ways that no ordinary person can understand. The very pick of the profession, both for probity and for efficiency, should have been chosen for the posts at the White City recruiting centre, instead of a pair of obvious failures such as these. In view of the revelations made, it is to be hoped that every exemption and postponement based upon certificates signed by Caley and Dow will be reviewed at once by competent officials.

Lastly, the medical profession will note that one of the two medical defendants is of military age, and should, therefore, long ago have come under the Service Acts and the Central Medical War Committee. It may not have been the fault of the latter, if his name was not supplied; but, on the face of matters, it seems as if a leakage had occurred somewhere. In which connection it may be worth adding that there is a good deal of exasperation in the profession at the slowness with which eligible young doctors from the Harley Street region are "combed out" by the special body which deals with them, compared with the energy with which all others in the profession are hunted down. If it is true, as rumoured, that those who have already served a year with temporary commissions are to be conscripted afresh shortly for another year of service, dissatisfaction is likely to be more vocal than it has been hitherto, for everyone in London institutional circles is wondering how some dozens, if not hundreds, of eligibles from Harley Street have contrived so far to avoid being called up.

